# Prevalence and predictors of depression, anxiety, and stress among refugees in Egypt

**DOI:** 10.1186/s42506-024-00158-8

**Published:** 2024-06-06

**Authors:** Engy M. El-Ghitany, Ayat Ashour, Ehab Elrewany, Azza G. Farghaly, Zeinab Shata

**Affiliations:** 1 Department of Tropical Health, High Institute of Public Health, Alexandria University, Alexandria, Egypt; 2 Department of Family Health, High Institute of Public Health, Alexandria University, Alexandria, Egypt

**Keywords:** Depression, Anxiety, Stress, Refugees, Mental problems, Egypt

## Abstract

**Background:**

Many Refugees show multiple distressing psychological and mental health problems associated with stressful and adverse conditions in host countries. Even though Egypt is one of the top five destination countries hosting refugees and asylum-seekers in the MENA region, there is a lack of studies investigating mental health problems among refugees in Egypt. This study aimed to assess the prevalence and predictors of depression, anxiety, and stress symptoms among refugees living in Egypt.

**Methods:**

A total of 398 refugees aged 18 and above were interviewed from migrants’/refugees' community centers in Giza, Alexandria, Dakahlia, and Damietta governorates. A predesigned structured interview questionnaire was used to collect socio-demographic and migration-related variables. The Arabic Version of Depression Anxiety Stress Scales (DASS-21) was used to assess the presence and severity of the three negative emotional states.

**Results:**

Nearly two-thirds of the participants were Syrian (66.4%). The majority resided in Alexandria and Giza governorates (75.9%), were females (73.4%), and were married (71.1%). The most reported migration difficulty was housing (41.5%). Anxiety symptoms were reported among 63.3% (95% CI: 58.59%- 68.05%) of sampled refugees, followed by depression (61.3%, 95% CI: 56.52%-66.10%), and stress symptoms (51.5%, 95% CI: 46.56%-56.36%). Results of regression analysis showed that; female gender predicted anxiety (aOR 2.419, 95% CI: 1.485–3.941, *p* < 0.001) and stress (aOR 2.053, 95% CI: 1.197–3.519, *p* = 0.009), while younger age groups (18–25 yr. and 26–50 yr.) compared to the older age group (51 yr. and older) predicted depression (aOR 4.529, 95% CI: 1.696–12.097, *p* = 0.003 and aOR 2.177, 95% CI: 1.270- 3.733, *p* = 0.005, respectively) and stress (aOR 4.334, 95% CI: 1.556–12.074, *p* = 0.005 and aOR 1.808, 95% CI: 1.023–3.193, *p* = 0.041, respectively). Residence in Alexandria, housing, and employment difficulties predicted anxiety (aOR 2.354, 95% CI: 1.420–3.902, *p* < 0.001, aOR 1.726, 95% CI: 1.073–2.778, *p* = 0.025, and aOR 3.044, 95% CI: 1.248–7.425, *p* = 0.014, respectively), depression (aOR 1.949, 95% CI: 1.163–3.266, *p* = 0.011, aOR 1.666, 95% CI: 1.036–2.681, *p* = 0.035, and aOR 3.216, 95% CI: 1.321–7.828, *p* = 0.010, respectively), and stress (aOR 2.450, 95% CI: 1.431–4.195, *p* < 0.001, aOR 1.911, 95% CI: 1.187–3.078, *p* = 0.008, and aOR 4.482, 95% CI: 1.867–10.760, *p* < 0.001, respectively).

**Conclusion:**

Mental health problems are prevalent among the refugee population in Egypt that are compounded by the difficult post-migration socio-economic situation. Multisectoral attention towards refugees’ mental health is urgently needed.

## Introduction

The refugee crisis has been escalating and gaining global attention as an increasing public health problem. Egypt remains one of the largest five destination countries hosting refugees and asylum-seekers in the MENA region [1–4]. In 2019, 254,726 refugees and asylum-seekers from 58 nationalities were registered in Egypt by the United Nations High Commissioner for Refugees (UNHCR) [5]. Syrian refugees remain the largest population among them, where they constitute nearly 50% of the total refugee and asylum seeker population since the huge, forced displacement of the Syrian population following the armed conflict that happened in Syria in 2011. Other nationalities of refugees include mainly Sudanese, Somalis, Iraqis, Ethiopians, South Sudanese, Eritreans, Yemenis, and Palestinians [6]. In Egypt, refugees do not live in camps but are living among Egyptian communities, mostly in urban cities like Alexandria, Cairo, Giza, and Qalyubia governorates [6]. It is worth mentioning that they have access to health services on equal footing to Egyptians, which represents an added challenge for the Egyptian economy [1].

Refugees are exposed to several stressors, including not only those related to war such as destruction of their homes, death of family members, torture, and leaving their homeland for an unknown future but in addition to their traumatic experiences in their mother countries, they are also affected by adverse circumstances in the host countries, where the ability for self-help and mutual support has been diminished [6–8].

It is estimated that 67% of Syrian refugees in Egypt are extremely vulnerable and in need of help. Several factors and challenges are involved in increasing vulnerability among refugees in Egypt. These factors include increases in living costs, lengthy procedures for residency renewals, facing difficulties in livelihood opportunities and access to financial services, the presence of administrative and legal barriers to formal employment as well as risks related to informal employment that can be sometimes unsafe and exploitative for the refugee households. Difficulties in meeting basic needs are one of the biggest challenges encountered by refugees in Egypt [1]. As a result, many refugees show multiple distressing psychological and somatic symptoms and mental health problems associated with stressful and adverse conditions [6, 9].

Epidemiological studies indicate that refugees are at substantial risk of developing symptoms of common mental disorders including depression, anxiety, post-traumatic stress disorder (PTSD) as well as psychosomatic symptoms [9–14]. It was estimated that the age-standardized point prevalence of major depression and PTSD in populations affected by conflicts is 7.6% and 12.9%, respectively [15]. Lack of social integration has been highly correlated with the occurrence of mental health problems among refugees, the severity of depression and anxiety symptoms, and decreased health-related quality of life. Meanwhile, symptoms of psychological distress among refugees predicted difficulties in social integration [16].

In light of the high rates of mental health problems among refugees, the multiple vulnerability factors and stressors they face in Egypt, and the burden such health problems put on the healthcare system in Egypt, addressing the mental health issues of refugees living in Egypt is of public health importance. Taking into consideration the lack of studies documenting the prevalence of mental health problems and disorders among refugees during their displacement and resettlement in Egypt, this research aimed to estimate the prevalence and predictors of mental health problems (Depression, anxiety, and stress) among refugees living in Egypt, and to determine associated factors. Providing data about the magnitude of the mental health problems among refugees living in Egypt will pave the way for cost-effective healthcare decisions taken by policymakers.

## Methods

### Study design and settings

A cross-sectional study was conducted in different Syrian and migrants’/refugees' community centers (non-governmental centers) in Giza, Alexandria, Dakahlia, and Damietta governorates. These centers provide services and activities for refugees such as women empowerment, workshops on how to run a business and improve marketing skills, educational sessions for children, and awareness sessions on public concern topics such as health and law topics. The study was conducted from October 2021 to January 2022. Each center was visited one to three times during the 15-week study duration. The day of the visit was selected randomly, and all eligible visitors on that day were invited to participate. Nearly two-thirds (66.3%) of the participant refugees were Syrian and the remaining were from Sudan, Eritrea, Ethiopia, Yemen, and Jordan.

### Sample size

The sample size was calculated based on the results of a previous meta-analysis [17], which reported a 15.6% prevalence of depression among migrants. A minimum sample size of 384 subjects was required with a margin of error of 0.04 and a 95% confidence level. The sample size was calculated using Epi INFO, Version 7.

### Target population

Refugees of both genders were recruited by inviting all those attending any of the study settings during the data collection days. Migrants who fled to Egypt before 2011, and migrants younger than 18 years old were excluded. A total sample of 398 refugees was obtained, with a response rate of 100 percent.

### Data collection tools

The survey was conducted using a predesigned structured interview questionnaire and included socio-demographic and migration-related variables, as well as the Arabic Version of Depression Anxiety Stress Scales (DASS-21) [18, 19] which is a set of 3 self-report scales designed to assess the presence and severity of three negative emotional states of depression, anxiety, and stress. Each of the three DASS-21 subscales contains 7 items to be responded to by a 4-point Likert scale ranging from 0 (not at all) to 3 (very much or most of the time) to rate the extent to which they have experienced each state over the past week. Scores for depression, anxiety, and stress are calculated by summing the scores for the relevant items and multiplying them by two. Depression, anxiety, and stress symptoms are identified at cut-off scores of 10, 8, and 15, respectively. The Arabic version of the DASS-21 has been demonstrated to be a valid and reliable screening instrument [18–20]. Cronbach's α values were used as a measure of internal consistency of DASS-21; the calculated Cronbach's α of depression, anxiety, and stress sections were 0.873, 0.829, and 0.889, respectively and Cronbach's α of the whole scale was 0.945.

Scores of the three DASS-21 subscales were interpreted as follows:

**Table:** 

	**Depression (D)**	**Anxiety (A)**	**Stress (S)**
**Normal**	0–9	0–7	0–14
**Mild**	10–13	8–9	15–18
**Moderate**	14–20	10–14	19–25
**Severe**	21–27	15–19	26–33
**Extremely severe**	28 +	20 +	34 +

The questionnaire was tested on a pilot sample of 15 refugees to check its wording and the clarity of questions. The questionnaire was clear and understandable with no additional modifications. The pilot sample was included in the recruited sample of refugees.

### Statistical analysis

Data were extracted, revised, coded, and fed to statistical software IBM SPSS version 25 (SPSS, Inc. Chicago, IL). Qualitative data were described using frequency and percentage while quantitative data were described using mean, and standard deviation. The Chi-square test, Fisher exact test, and Monte Carlo correction were used to test the association between categorical variables. Three multiple logistic regression analyses with stepwise backward methods were conducted to explore the association of socio-demographic and migration-related variables with depression, anxiety, and stress. Depression, anxiety, and stress symptoms were identified at cut-off scores of 10, 8, and 15, respectively, and coded as binary variables (0 and 1). All independent variables (13 variables) in the bivariate analysis were entered into the regression model. The significance level in all analyses was predetermined at *p* ≤ 0.05. All statistical analyses were done using two-tailed tests.

## Results

### Characteristics of the sample

The sampled refugees aged from 18–85 years old, with a mean of 40.97 ± 13.66 years. Most participants were female refugees (73.4%) and married (71.1%). One-third of them had secondary education (30.2%), followed by university graduates (28.9%). Regarding the occupation, more than half of them were not working (65.1%), including housewives (52.5%), while students constituted only 8% of the sample. Most of the sampled refugees (75.9%) reside in Alexandria and Giza Governorates. Meanwhile, 38.9% of the sample reported insufficient monthly income and debt. More than half of the participants (53.3%) lived in Egypt for more than 5 years, while only 5% came to Egypt within one year. The most reported difficulties were housing (41.5%) followed by access to educational services (16.8%) A history of chronic diseases was reported among nearly half of the sampled refugees (57.3%) (Table 1).

**Table 1:** Sociodemographic and migration-related characteristics of the studied refugees, Egypt 2021–2022 (*n* = 398)

Characteristics	Total (*n* = 398)	
	No.	%
**Age (years)**		
18–25	67	16.8
26–50	237	59.5
51 +	94	23.7
Min–Max	18–85	
Mean ± SD	40.97 ± 13.66	
**Gender**		
Male	106	26.6
Female	292	73.4
**Marital status**		
Single	72	18.1
Married	283	71.1
Divorced/ Widowed	43	10.8
**Education**		
Primary	73	18.3
Preparatory	90	22.6
Secondary	120	30.2
University	115	28.9
**Occupation**		
Student	32	8.0
Housewife	209	52.5
Not working	50	12.6
Non-professional job	64	16.1
Professional job	43	10.8
**Current residence**		
Alexandria	176	44.2
Dakahlia	45	11.3
Damietta	51	12.8
Giza	126	31.7
**Monthly income**		
Sufficient	90	22.6
Insufficient	153	38.4
Insufficient and in debt	155	39.0
**Duration of living in Egypt (Years)**		
less than 1 year	20	5.0
1–5	166	41.7
More than 5 years	212	53.3
**Housing difficulties**		
No	233	58.5
Yes	165	41.5
**Employment difficulties**		
No	354	88.9
Yes	44	11.1
**Difficulties to access educational services**		
No	331	83.2
Yes	67	16.8
**Difficulties to access health services**		
No	372	93.5
Yes	26	6.5
**History of chronic diseases (comorbidities)**^**a**^		
No	170	42.7
Yes	228	57.3

^a^Comorbidities include hypertension, diabetes mellitus, chronic kidney diseases, COPD, cancer, cardiovascular diseases, and chronic liver diseases

### Prevalence of depression, anxiety, and stress symptoms among sampled refugees

Anxiety symptoms were reported among 63.3% of sampled refugees, followed by depression symptoms (61.3%), and stress symptoms were the least (51.5%). Regarding the severity of symptoms, 36.6% reported severe and extremely severe anxiety symptoms, followed by 28.9% severe and extremely severe stress symptoms, and 25.9% severe and extremely severe depression symptoms (Table 2).

**Table 2:** Prevalence and severity of depression, anxiety, and stress symptoms (DASS-21) among studied refugees, Egypt, 2021–2022 (*n* = 398)

Prevalence and severity	Depression		Anxiety		Stress	
	**No.**	**%**	**No.**	**%**	**No.**	**%**
**Normal**	154	38.7	146	36.7	193	48.5
**Mild**	55	13.8	26	6.5	44	11.1
**Moderate**	86	21.6	80	20.1	46	11.6
**Severe**	39	9.8	36	9.0	64	16.1
**Extremely severe**	64	16.1	110	27.6	51	12.8
**Prevalence% (95% CI)**	61.3 (95% CI: 56.52- 66.10)		63.3 (95% CI: 58.59- 68.05)		51.5 (95% CI: 46.56- 56.36)	
Min–Max	0–42		0–42		0–42	
Mean ± SD	14.20 ± 11.146		12.92 ± 10.270		17.15 ± 11.92	

### Factors associated with depression, anxiety, and stress among sampled refugees

Female refugees reported significantly higher symptoms of anxiety compared to male refugees (68.8% vs. 48.1%, *p* < 0.001). The governorate of current residence was significantly associated with symptoms of anxiety, where Alexandria refugees had the highest rate of anxiety (73.3%, *p* < 0.001). Insufficiency of the monthly income and being in debt, facing housing and employment difficulties, having difficulties accessing educational services, and having comorbid diseases were significantly associated with symptoms of anxiety (*p* ≤ 0.05).

Divorced and widowed refugees had a higher rate of depression (76.7%, *p* = 0.026) and similar to anxiety, Alexandria refugees exhibited the highest rate of depression (69.9%%, *p* < 0.001). Insufficiency of the monthly income and being in debt, facing housing and employment difficulties, and having difficulties accessing educational services, were significantly associated with symptoms of depression (*p* ≤ 0.05).

Similar to anxiety, female refugees reported significantly higher symptoms of stress compared to male refugees (56.8% vs. 36.8%, *p* < 0.001). Also, the governorate of current residence was significantly associated with symptoms of stress, where Alexandria refugees had the highest rate of stress (60.2%, *p* = 0.003). Insufficiency of monthly income being in debt, facing housing and employment difficulties, and having difficulties accessing educational services were significantly associated with symptoms of stress (*p* ≤ 0.05) (Table 3).

**Table 3:** Prevalence of depression, anxiety, and stress according to some sociodemographic and migration-related characteristics among studied refugees, Egypt, 2021–2022 (*n* = 398)

Characteristics	**Anxious (score > 7)**		***p*-value**	**Depressed (score > 9)**		***p*-value**	**Stressed (Score > 14)**		***p*-value**
	No.	%		No.	%		No.	%	
**Age (years)**									
18–25	42	62.7	0.243	40	59.7	0.079	35	52.2	0.077
26–50	157	66.2		155	65.4		131	55.3	
51 +	53	56.4		49	52.1		39	41.5	
**Gender**									
Male	51	48.1	< 0.001*	57	53.8	0.063	39	36.8	< 0.001*
Female	201	68.8		187	64.0		166	56.8	
**Marital status**									
Single	40	55.6	0.125	37	51.4	0.026*	31	43.1	0.227
Married	180	63.6		174	61.5		149	52.7	
Divorced/Widowed	32	74.4		33	76.7		25	58.1	
**Education**									
Primary	44	60.3	0.061	44	60.3	0.150	36	49.3	0.276
Preparatory	67	74.4		64	71.1		54	60.0	
Secondary	68	56.7		72	60.0		56	46.7	
University	73	63.5		64	55.7		59	51.3	
**Occupation**									
Student	18	56.3	0.072	16	50.0	0.083	13	40.6	0.281
Housewife	146	69.9		139	66.5		117	56.0	
Not working	27	54.0		27	54.0		21	42.0	
Non-professional job	35	54.7		41	64.1		32	50.0	
Professional job	26	60.5		21	48.8		22	51.2	
**Current residence**									
Alexandria	129	73.3	< 0.001*	123	69.9	< 0.001*	106	60.2	0.003*
Dakahlia	20	44.4		18	40.0		20	44.4	
Damietta	35	68.6		32	62.7		29	56.9	
Giza	68	54.0		71	56.3		50	39.7	
**Monthly income**									
Sufficient	46	51.1	0.008*	44	48.9	0.017*	36	40.0	< 0.001*
Insufficient	96	62.7		96	62.7		70	45.8	
Insufficient and in debt	110	71.0		104	67.1		99	63.9	
**Duration of living in Egypt (Years)**									
less than 1 year	9	45.0	0.210	12	60.0	0.797	5	25.0	0.052
1–5	108	65.1		105	63.3		88	53.0	
More than 5 years	135	63.7		127	59.9		112	52.8	
**Housing difficulties**									
No	132	56.7	< 0.001*	126	54.1%	< 0.001*	100	42.9	< 0.001*
Yes	120	72.7		118	71.5%		105	63.6	
**Employment difficulties**									
No	215	60.7	0.002*	207	58.5%	< 0.001*	169	47.7	< 0.001*
Yes	37	84.1		37	84.1%		36	81.8	
**Difficulties to access educational services**									
No	202	61.0	0.035*	195	58.9	0.029*	161	48.6	0.011*
Yes	50	74.6		49	73.1		44	65.7	
**Difficulties to access health services**									
No	231	62.1	0.056	225	60.5	0.202	187	50.3	0.061
Yes	21	80.8		19	73.1		18	69.2	
**Comorbidities**^**a**^									
No	102	60.0	0.049*	97	57.1	0.133	78	45.9	0.053
Yes	150	65.8		147	64.5		127	55.7	

^a^Comorbidities include hypertension, diabetes mellitus, chronic kidney diseases, COPD, cancer, cardiovascular diseases, and chronic liver diseases

^*^ Significant *at p* value ≤ 0.05

### Results of logistic regression analysis

According to Table 4, predictors of anxiety were female gender (aOR 2.419, 95% CI: 1.485–3.941, *p* < 0.001), residence in Alexandria (aOR 2.354, 95% CI: 1.420–3.902, *p* < 0.001), housing difficulties (aOR 1.726, 95% CI: 1.073–2.778, *p* = 0.025) and employment difficulties (aOR 3.044, 95% CI: 1.248–7.425, *p* = 0.014).

**Table 4:** Results of stepwise logistic regression analysis of variables associated with anxiety, depression, and stress among studied refugees (*n* = 398)

Variables	**B**	**SE**	***p*-value**	**aOR**	**95% CI for aOR**	
					**LL**	**UL**
**Anxiety**						
**Gender**						
Male (Ref.)						
Female	0.883	0.249	< 0.001*	2.419	1.485	3.941
**Current residence**						
Alexandria	0.856	0.258	< 0.001*	2.354	1.420	3.902
Dakahlia	-0.081	0.371	0.827	0.922	0.446	1.906
Damietta	0.713	0.370	0.054	2.041	0.988	4.213
Giza (Ref.)						
**Housing difficulties**						
No (Ref.)						
Yes	0.546	0.243	0.025*	1.726	1.073	2.778
**Employment difficulties**						
No (Ref.)						
Yes	1.113	0.455	0.014*	3.044	1.248	7.425
**Depression**						
**Age (years)**						
18–25	1.511	0.501	0.003*	4.529	1.696	12.097
26–50	0.778	0.275	0.005*	2.177	1.270	3.733
51 + (Ref.)						
**Marital status**						
Single (Ref.)						
Married	1.040	0.424	0.014*	2.828	1.233	6.486
Divorced/Widowed	2.069	0.571	< 0.001*	7.917	2.584	24.256
**Current residence**						
Alexandria	0.667	0.263	0.011*	1.949	1.163	3.266
Dakahlia	-0.449	0.384	0.242	0.638	0.301	1.354
Damietta	0.368	0.372	0.322	1.445	0.697	2.993
Giza (Ref.)						
**Housing difficulties**						
No (Ref.)						
Yes	0.511	0.243	0.035*	1.666	1.036	2.681
**Employment difficulties**						
No (Ref.)						
Yes	1.168	0.454	0.010*	3.216	1.321	7.828
**Comorbidities**						
No (Ref.)						
Yes	0.506	0.239	0.034*	1.658	1.038	2.649
**Stress**						
**Age (years)**						
18–25	1.467	0.523	0.005*	4.334	1.556	12.074
26–50	0.592	0.290	0.041*	1.808	1.023	3.193
51 + (Ref.)						
**Gender**						
Male (Ref.)						
Female	0.719	0.275	0.009*	2.053	1.197	3.519
**Marital status**						
Single (Ref.)						
Married	0.993	0.444	0.025*	2.700	1.131	6.446
Divorced/Widowed	1.424	.574	0.013*	4.155	1.348	12.809
**Current residence**						
Alexandria	0.896	0.274	< 0.001*	2.450	1.431	4.195
Dakahlia	0.641	0.405	0.114	1.898	.858	4.201
Damietta	0.836	0.383	0.029*	2.307	1.089	4.887
Giza (Ref)						
**Monthly income**						
Sufficient (Ref.)						
Insufficient	0.254	0.294	0.389	1.289	0.724	2.295
Insufficient and in debt	0.677	0.301	0.025*	1.969	1.091	3.553
**Housing difficulties**						
No (Ref.)						
Yes	0.648	0.243	0.008*	1.911	1.187	3.078
**Employment difficulties**						
No (Ref.)						
Yes	1.500	0.447	< 0.001*	4.482	1.867	10.760
**Comorbidities**^**a**^						
No (Ref.)						
Yes	0.595	0.243	0.014*	1.813	1.126	2.919

*SE* standard error, *CI* confidence interval

^a^Comorbidities include hypertension, diabetes mellitus, chronic kidney diseases, COPD, cancer, cardiovascular diseases, and chronic liver diseases

^*^Significant *p* value ≤ 0.05

Younger age groups (18–25 and 26–50) had higher odds of depression compared to older group (51 years and older); (aOR 4.529, 95% CI: 1.696–12.097, *p* = 0.003 and aOR 2.177, 95% CI: 1.270- 3.733, *p* = 0.005, respectively). Housing and employment difficulties predicted symptoms of depression (aOR 1.666, 95% CI: 1.036–2.681, *p* = 0.035 and aOR 3.216, 95% CI: 1.321–7.828, *p* = 0.010, respectively). The presence of comorbidities was independently associated with depression symptoms (aOR 1.658, 95% CI: 1.038–2.649, *p* = 0.034). Similar to anxiety, residence in Alexandria predicted depression symptoms (aOR 1.949, 95% CI: 1.163–3.266, *p* = 0.011).

Compared to single refugees, being married, or divorced/widowed increased the odds of depression (aOR 2.828, 95% CI: 1.233–6.486, *p* = 0.014 and aOR 7.917, 95% CI: 2.584–24.256, *p* < 0.001, respectively). Similarly, married and divorced/widowed refugees had higher odds of stress (aOR 2.700, 95% CI: 1.131- 6.446, *p* = 0.025 and aOR 4.155, 95% CI: 1.348–12.809, *p* = 0.013, respectively).

The female gender was a predictor for stress symptoms (aOR 2.053, 95% CI: 1.197–3.519, *p* = 0.009) among the sampled refugees. Similar to depression, younger age groups (18–25 and 26–50) had higher odds of stress compared to the older group (51 years and older); (aOR 4.334, 95% CI: 1.556–12.074, *p* = 0.005 and aOR 1.808, 95% CI: 1.023–3.193, *p* = 0.041, respectively). Also, housing and employment difficulties predicted symptoms of stress (aOR 1.911, 95% CI: 1.187–3.078, *p* = 0.008 and aOR 4.482, 95% CI: 1.867–10.760, *p* < 0.001, respectively). The presence of comorbidities was independently associated with stress symptoms (aOR 1.813, 95% CI: 1.126–2.919, *p* = 0.014). Like anxiety and depression, residence in Alexandria predicted stress symptoms (aOR 2.450, 95% CI: 1.431–4.195, *p* < 0.001). Moreover, having insufficient monthly income was independently associated with stress symptoms (aOR 1.969, 95% CI: 1.091–3.553, *p* = 0.025) (Table 4).

## Discussion

The present study investigated mental health problems (anxiety, depression, and stress) among a sample of refugees living in Egypt. Anxiety, depression, and stress symptoms of variable severity were reported among nearly half of the sampled refugees. Interestingly, severe, and extremely severe symptoms among sampled refugees were highest for anxiety (36.6%), followed by stress (28.9%), and least for depression (25.9%).

These rates were lower than the rate of depression (63%) and much lower for anxiety (89%) revealed by a study conducted on Syrian refugees in Cairo (2020) [21]. Compared to the prevalence estimates of depression and anxiety among Egyptians (5% and 6%, respectively) reported by the Global Burden of Disease study (2019) [22], the aforementioned rates indicate a high magnitude of the problem among refugees.

Refugees’ mental health has been investigated in many studies with different populations, different hosting cultures, and countries, however, the findings were heterogeneous and inconsistent, making it difficult to estimate the prevalence of mental health problems in these populations [5, 9, 23–27]. Systematic reviews revealed large variations in reported prevalence rates of depression (5%-80%), and PTSD (3%-88%) [28, 29]. In their meta-analysis, Fazel et al. (2005) reported a prevalence of 4–6% for depression (14 studies) and 8–10% for PTSD (17 studies) among adult refugees [11]. Two more recent meta-analyses [14, 30], described considerably higher prevalence rates (25–45% for depression, 21–35% for anxiety, and 31–63% for PTSD). Henkelmann et al. (2020) [12] explained this difference as a result of the inclusion of both diagnostic interviews and self-report assessment tools in the latter two meta-analyses [14, 30], while in Fazel et al. meta-analysis [11], only studies in which mental health was assessed by diagnostic interviews were included [12]. In an earlier systematic review (2015), Bogic et al. [13] concluded that the observed heterogeneity was attributed markedly to the clinical and methodological factors, the mother country of the refugees, and the host country.

The comorbidity between depression, anxiety, and stress was evident in the current study, compounding the impact of high prevalence rates of each of the three psychological states among refugees living in Egypt.

In the current study, socio-demographic predictors of mental health problems among refugees living in Egypt included the female gender and having insufficient monthly income and being in debt. Evidence for gender differences similar to current findings, and poor socio-economic status were reported in other studies among refugees and immigrants [10, 14, 31–33]. Other socio-demographic predictors included younger age groups and married, divorced, or widowed refugees. A shared explanation for such findings could be related to the nature of stressors faced by these groups of refugees, precisely including those stressors related to taking care of a family, parenting responsibilities, and livelihood demands that are faced more by people in the younger reproductive age groups and those who are married, divorced or widowed in comparison to elderly and single ones.

The association between living in Alexandria and the anxiety symptoms can be explained considering two factors: firstly, Alexandria hosted almost half of the sampled refugees; and secondly, it is considered the second capital of Egypt where the lifestyle is full of challenges and stressors compared to other governorates included in the study which are considered less urban than Alexandria governorate. Moreover, the highest prevalence rate of anxiety symptoms especially the severe and extremely severe symptoms compared to depression could provide an additional explanation.

On the other hand, environmental factors encountered in Egypt as predictors of mental health problems included housing problems and employment difficulties. Similarly, a growing number of studies in the host countries found that stressors and conditions of adversity, including economic hardship, lack of employment and educational opportunities, restricted access to services, insecure housing, and residency are among the determinants of poor mental health among refugees [14, 31–33].

In addition, physical health was an important predictor of refugees’ mental health; the presence of comorbid chronic diseases predicted mental health problems among sampled refugees. In consistency with this finding, similar studies among Syrian refugees in Jordan confirmed the relationship between chronic diseases and mental health problems [23, 34].

### Limitations of the study

The findings of the current study should be interpreted in light of some limitations such as using self-reported tools to assess depression, anxiety, and stress symptoms among refugees rather than interviews, which would give a more specific and accurate estimation of the prevalence of these mental health problems. However, conducting diagnostic clinical interviews was not feasible in the settings where the sample of refugees was recruited. Further large-scale studies are needed to give more comprehensive data on the mental health profile of refugees living in Egypt. Moreover, the elevated heterogeneity of the sample constituted another limitation, as well as the cross-sectional study design, which precludes establishing a causal association.

## Conclusion

The current work revealed that the prevalence and comorbidity of mental health problems, including anxiety, depression, and stress is high among the refugee population in Egypt. Psychological distress among refugees is compounded by the difficult post-migration socio-economic situation, where they face housing and employment difficulties and economic hardship in their monthly income. In addition, being a woman refugee increases the risk of developing mental health problems.

A comprehensive response is urgently needed from multiple sectors, including the relevant government sectors and in particular, the healthcare system, and the community sectors, to address the refugees' mental health needs, reduce post-migration stressors, and provide needed mental health services. The clinical implications include providing different interventions including screening programs in the primary health care facilities for early identification and treatment, as well as providing evidence-based psychosocial interventions for at-risk groups, and referral services for severe cases.

Future studies on refugee mental health should assess the role of past traumatic experiences on refugees' mental health residing in Egypt and the relationship between symptoms of psychological distress and refugees' quality of life. In addition, future research should be directed to assess mental health care services provided to refugees in Egypt.

## Data Availability

The data are available from the corresponding author on reasonable request.

## Abbreviations

aOR: Adjusted Odds Ratio
CI: Confidence Interval
DASS: Depression Anxiety Stress Scales
IRB: Institutional Review Board
MENA: The Middle East and North Africa
OR: Odds Ratio
PTSD: Post-Traumatic Stress Disorder
SPSS: Statistical Packages for Social Sciences
SD: Standard Deviation
SE: Standard Error
UNHCR: United Nations High Commissioner for Refugees
